# The effects of horticultural therapy on the functionality of psychotic patients employed in the green unit of the psychiatric hospital of thessaloniki greece

**DOI:** 10.1192/j.eurpsy.2021.1354

**Published:** 2021-08-13

**Authors:** K. Kontos, S. Koutsou, A. Sismanidi, N. Theodoropoulou, V. Nikolopoulou, M. Filippiadou, G. Papazisis

**Affiliations:** 1 Green Unit, Psychiatric Hospital of Thessaloniki, Thessaloniki, Greece; 2 School Of Agriculture, Department Of Agricultural Economics, International Hellenic University, Thessaloniki, Greece; 3 School Of International Studies Communication And Culture, Panteion University of Social and Political Sciences, Athens, Greece; 4 School Of Medicine, Aristotle University of Thessaloniki, THESSALONIKI, Greece

**Keywords:** Horticultural therapy, Rehabilitation, Functionality, schizophrénia

## Abstract

**Introduction:**

Horticultural therapy is used as alternative treatment option in rehabilitation programs for patients suffering from chronic mental disorders. In Greece, the Psychiatric Hospital of Thessaloniki was the first that organized a organized Rehabilitation Program approved by the Greek Ministry of Health, the “Green Unit - Monada Prasinou”.

**Objectives:**

The purpose of this study was to investigate the effect of horticultural intervention on the functionality of patients employed in the Green Unit of the Psychiatric Hospital of Thessaloniki.

**Methods:**

The Mini-ICF-APP Social Functioning Scale was used to evaluate the functionality of the patients. Horticulture therapy included actual gardening and other agricultural activities. The sample consisted of two groups, 22 inpatient of the Green Unit and 22 patients of outpatient units as a control group. The majority of the patients suffered from Schizophrenia spectrum disorders. Both patients’ groups were evaluated over 12 months (May 2018- May 2019).

**Results:**

At baseline evaluation, the patients of the Green Unit patients presented higher scores only in the mobility and endurance subscale. A year later there was a statistically significant difference in all thirteen subscales. Shortly, the patients were rated as more consistent, organizational, social, flexible, responsible, more confident and enterprising, more adaptable and more able to take care of themselves.

**Conclusions:**

The results suggest that horticulture as a therapeutic method had beneficial effects in all dimensions of patient functionality confirming the hypothesis that such programs should be a priority in the effort of psychosocial reintegration of patients suffering from chronic mental disorders.

